# Bone Development and Growth

**DOI:** 10.3390/ijms25126767

**Published:** 2024-06-20

**Authors:** José M. López

**Affiliations:** Department of Morphology and Cellular Biology, Faculty of Medicine, University of Oviedo, 33006 Oviedo, Spain; jmlopez@uniovi.es

Our skeleton is an essential part of our body consisting of 206 pieces made of a specialized form of connective tissue, with a matrix containing collagen fibers and a large amount of minerals. Collagen fibers give the tissue tensile strength and resistance to stress, whereas the inorganic matrix, composed of a combination of calcium and phosphorus salts, contributes to its hardness and rigidity. The dense, strong, and hardy nature of bone tissue allows it to form a framework that supports all the organs of the body and provides safe shelter for the most vital and delicate of them, such as the brain, heart, and lungs. Although hardness and rigidity are key bone structural attributes that determine specific properties of this organ, the skeleton is much more than a rigid scaffold for the body. It is a multifunctional organ that transforms the force created by muscle contraction into locomotion, contains the marrow where the blood cells are produced, serves as the main storage system for calcium and phosphorus that are key components of intracellular signaling pathways, is an essential part of the circulatory system, and has emerged in the last years as a previously unsuspected endocrine tissue [1,2].

Bone growth is not only a critical process for body development and growth but also a challenge per se, since hardness and rigidity are properties normally incompatible with the high plasticity necessary for the growth of the tissues. A bone is an inextensible structure; so, new elements in a growing bone only can be only incorporated appositionally from successive layers on a free surface. This fact would restrict the speed of growth; however, bones have evolved special mechanisms that allow not only extensive longitudinal growth but also compatible growth with the maintenance of adequate mechanical properties for supportive function [3,4]. On this basis, it was the aim of this Special Issue to collect original research and review articles that illustrate and encourage the attempts to increase our understanding of the cellular and molecular mechanisms underlying bone development from an interdisciplinary perspective.

Bone formation is classically considered to occur through two different mechanisms: direct: intramembranous ossification or indirect: endochondral ossification [5]. These two modes of ossification differ in some ways including the origin, timing, growth performance, and pattern of development. Intramembranous ossification is a more limited type of ossification used for the development of the flat bones of the face, most of the cranial bones, and the clavicles, whereas endochondral ossification generates the majority of axial and appendicular skeletal structures, including vertebral bodies and long bones, such as the femur and tibia. Intramembranous ossification is primarily initiated by proliferation of mesenchymal stem cells into high vascularized areas of embryonic connective tissue to form sheets of mesenchymal cells that form a template of the future bone. The same mesenchymal cells forming the template later directly differentiate into osteogenic cells that subsequently develop into early osteoblasts that secrete uncalcified matrix or osteoid that is subsequent mineralized by calcium and phosphate ions to produce hardened mineralized bone. Intramembranous ossification is considered to be more intense and lasts for less time than endochondral ossification; it begins during fetal development, and, by the time of birth, the bones formed by this process are much more advanced than those formed by endochondral ossification [6]. Nevertheless, neither the clavicles nor the skull and the skull sutures are fully ossified, and this allows these bones to deform during passage through the birth canal. Likewise, the flat bones of the face do not complete their intramembranous ossification until the end of the adolescent growth spurt.

Endochondral ossification implies a more complex process that also begins in the early embryo with the formation of a local condensation of mesenchymal cells that will constitute the primordia of the bones. Subsequently, most of these cells differentiate into chondrocytes, whereas a small number at the periphery remain as mesenchymal and form the perichondrium. As a result, a transient cartilaginous skeleton formed by a framework of cartilage templates with specific shapes is generated, and an increase in size occurs during the growth period. Afterward, chondrocytes in the central region of the template become hypertrophic, whereas some of the peripheral undifferentiated cells differentiate into osteoblasts that secrete osseous matrix and produce periosteal bone [7,8]. Then, periosteal buds containing blood vessels and perivascular mesenchymal cells invade the middle of the template to generate the primary ossification center, where cartilage begins to be replaced by bone. In addition to this invasion at the central region, secondary ossification centers are subsequently formed at each distal extreme of the template or epiphyses. The primary and secondary ossification centers remain separated by a thin layer of cartilage during the period of active bone growth, the so-called metaphyseal growth plate. The metaphyseal growth plate continuously produces new cartilage, and the replacement of cartilage by bone occurs at the ossification front. Cartilage resorption is balanced by continuous cartilage production, and, as a result, the amount of cartilage remains fairly constant, and a continuous increase in the osseous tissue is produced. After the adolescent growth spurt, all cartilage is replaced by osseous tissue; then, longitudinal bone growth ceases.

Intramembranous and endochondral ossification are usually considered as separate modes of ossification; however, this is a simplification, since it has been reported that both occur simultaneously during the development of many bones [9,10]. Furthermore, although it is assumed that long bones are formed by endochondral ossification, much of their postnatal ossification occurs subperiosteally and so is essentially intramembranous. In any case, bone formation always depends on the deposition of bone matrix by the osteoblasts, and it has not reported that different subpopulations of osteoblasts could contribute differently to intramembranous or endochondral ossification. In fact, osteoblasts require a scaffold that allow them to settle and deposit bone tissue; from a cellular point of view, differences in the type of ossification are differences in the type of scaffolds. In this way, it could be considered that there are types of scaffolds for osteoblasts to deposit bone: unmineralized tissue, hypertrophic-mineralized cartilage, and former bone. The first is associated with intramembranous ossification, the second with endochondral ossification, and the third with periosteal ossification. Very often, these three types of scaffolds are present in close regions of the same bone, and different ossification processes take place concurrently (Figure 1) [11].

A relevant process where intramembranous and endochondral ossification occur simultaneously is bone healing. Bones possess a high self-regenerative capacity; they have the ability to activate bone production following a fracture, and it is remarkable that the normal pathway of osteogenesis during development is recapitulated in bone healing [12,13]. The periosteum is critical for bone repair, since it is one of the main sources of mesenchymal stem cells that can migrate to the fractured bone region and differentiate depending on the local mechanical microenvironment. Mesenchymal cells can differentiate directly into osteoblasts and undergo direct bone formation, in a process that could be considered as intramembranous ossification. Likewise, mesenchymal cells differentiate into chondrocytes at the gap of the fracture to form a cartilaginous callus that bridges the fracture. These chondrocytes subsequently undergo maturation towards hypertrophy, express type X collagen, and the extracellular matrix is calcified and then degraded by proteases and invaded by vascular vessels and osteogenic cells. As a result, trabecular bone is formed by a process that could be considered endochondral ossification. Furthermore, the newly formed bone is remodeled into cortical bone through the coordinated actions of osteoblasts and osteoclasts.

A remarkable aspect of bone development is that the long-accepted idea that lineage-committed cells mature into end-stage differentiated cells that are programmed to die and to be replaced by newly differentiated cells arising from the same lineage pathway has been revised in recent years. Studies using lineage tracing techniques to follow the fate of chondrocytes in different bones during bone growth, as well as during bone fracture repair, have reported that a subset of chondrocytes can elude apoptosis and is able to transdifferentiate into osteoblasts at the chondro–osseous junction [14,15]. The emerging models of chondrocyte-to-osteoblast transdifferentiation open a new scenario of the basis of the well-known skeletal cell plasticity and provide an overall mechanism underlying many different cell type conversions, reported for a long time during the formation of osseous tissue in different bones. Furthermore, these results open a new scenario of possible innovative biomechanical strategies for the treatment of osteo-degenerative diseases.

In summary, bone biology is a multifaceted and strongly growing area of research that includes traditional fields, such as anatomy, physiology, biochemistry, and biomechanics, and also innovative approaches such as molecular genetics or tissue engineering. Although, in the last decade, progress has been made in our understanding of bone biology, many important questions still remain unanswered. Regulatory pathways in bone development are very complex, multidirectional, and multifaceted, with new results and perspectives regularly. It is beyond the scope of this review to discuss diverse aspects of bone development including the normal structure and cell biology in experimental models as well as the evaluation of pathological conditions that affect the skeleton. Well-documented reviews are included, and, overall, the current research trends in bone histology are discussed with a focus on new technological methods. The studies presented are interesting examples of the progressing bone science, which, using new experimental tools, can improve the understanding of the mechanisms underlying bone structure and function, and try to apply this new knowledge to the consequent improvement of new therapeutic methods to control tissue injury or even to progress regenerative medicine. There are still some clinical situations in which the process of bone regeneration is impaired, and the development of treatments that can be used for the enhancement of these complex clinical situations is of major relevance.

## Figures and Tables

**Figure 1:**
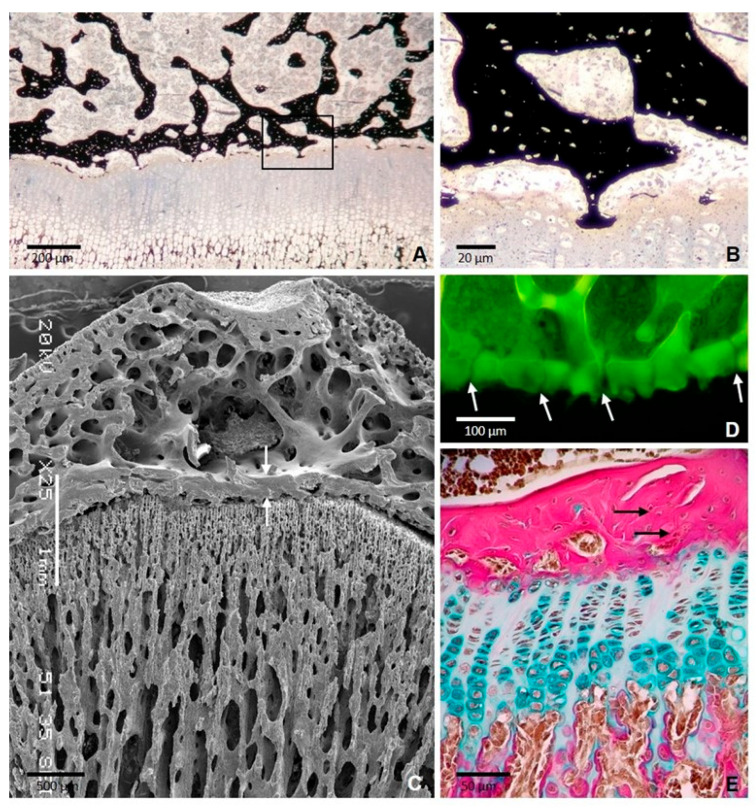
Formation of the epiphyseal bone plate in the tibial epiphysis of a 35-day-old rat. (**A**) Section stained with von Kossa showing thin bony extensions bridging the epiphyseal bone plate and the growth plate. The boxed area is shown at a higher magnification in (**B**) SEM image showing the epiphyseal plate (arrows) and the continuity of the bone plate with the trabecular meshwork of the epiphysis. (**C**) SEM image showing the epiphyseal plate (arrows) between the trabecular bone of epiphysis and diaphysis. (**D**) Confocal microscopy image of a 3D projection reconstructed from z-stack images showing the foramina of the plate through which blood vessels passed (arrows). (**E**) Paraffin section stained with Alcian blue/acid fuchsin showing that the collagen fibers are densely packed but are not arranged in a regular parallel pattern (arrows) [11].

## References

[1] Burr D.B., Akkus O., Burr D.B., Allen M.R. (2014). Bone Morphology and Organization. Basic and Applied Bone Biology.

[2] Hall B.K., Hall B.K. (2015). Bone. Bones and Cartilage.

[3] Shapiro F. (2016). Developmental Bone Biology. Pediatric Orthopedic Deformities.

[4] Xie M., Chagin A.S. (2021). The epiphyseal secondary ossification center: Evolution, development and function. Bone.

[5] Hunziker E.B. (1994). Mechanism of longitudinal bone growth and its regulation by growth plate chondrocytes. Microsc. Res. Techniq..

[6] Cohen M. (2000). Merging the old skeletal biology with the new. I. Intramembranous ossification, endochondral ossification, ectopic bone, secondary cartilage, and pathologic considerations. J. Craniofac. Genet. Dev. Biol..

[7] Rivas R., Shapiro F. (2002). Structural stages in the development of the long bones and epiphysis. J. Bone Jt. Surg..

[8] Cancedda R., Castagnola P., Cancedda F.D., Dozin B., Quarto R. (2000). Developmental control of chondrogenesis and osteogenesis. Int. J. Dev. Biol..

[9] Li X., Yang S., Jing D., Qin L., Zhao H., Yang S. (2020). Type II collagen-positive progenitors are major stem cells to control skeleton development and vascular formation. BioRxiv.

[10] Nagashima H., Sugahara F., Watanabe K., Shibata M., Chiba A., Sato N. (2016). Developmental origin of the clavicle, and its implications for the evolution of the neck and the paired appendages in vertebrates. J. Anat..

[11] Fernández-Iglesias A., Fuente R., Gil-Peña H., Alonso-Durán L., Santos F., López J.M. (2021). The Formation of the Epiphyseal Bone Plate Occurs via Combined Endochondral and Intramembranous-Like Ossification. Int. J. Mol. Sci..

[12] Gerstenfeld L.C., Cullinane D.M., Barnes G.L., Graves D.T., Einhorn T.A. (2003). Fracture healing as a post-natal developmental process: Molecular, spatial, and temporal aspects of its regulation. J. Cell. Biochem..

[13] Barnes G.L., Kostenuik P.J., Gerstenfeld L.C., Einhorn T.A. (1999). Growth factor regulation of fracture repair. J. Bone Miner. Res..

[14] Newton P.T., Li L., Zhou B., Schweingruber C., Hovorakova M., Xie M., Sun X., Sandhow L., Artemov A.V., Ivashkin E. (2019). A radical switch in clonality reveals a stem cell niche in the epiphyseal growth plate. Nature.

[15] Wong S.A., Hu D.P., Slocum J., Lam C., Nguyen M., Miclau T., Marcucio R.S., Bahney C.S. (2021). Chondrocyte-to-Osteoblast Transformation in Mandibular Fracture Repair. J. Orthop. Res..

